# The Natural Stilbenoid Piceatannol Decreases Activity and Accelerates Apoptosis of Human Neutrophils: Involvement of Protein Kinase C

**DOI:** 10.1155/2013/136539

**Published:** 2013-10-31

**Authors:** Viera Jancinova, Tomas Perecko, Radomir Nosal, Klara Svitekova, Katarina Drabikova

**Affiliations:** ^1^Institute of Experimental Pharmacology and Toxicology, Slovak Academy of Sciences, Dúbravská cesta 9, 841 04 Bratislava, Slovakia; ^2^National Transfusion Service, Limbová 3, 831 01 Bratislava, Slovakia

## Abstract

Neutrophils are able to release cytotoxic substances and inflammatory mediators, which, along with their delayed apoptosis, have a potential to maintain permanent inflammation. Therefore, treatment of diseases associated with chronic inflammation should be focused on neutrophils; formation of reactive oxygen species and apoptosis of these cells represent two promising targets for pharmacological intervention. Piceatannol, a naturally occurring stilbenoid, has the ability to reduce the toxic action of neutrophils. This substance decreased the amount of oxidants produced by neutrophils both extra- and intracellularly. Radicals formed within neutrophils (fulfilling a regulatory role) were reduced to a lesser extent than extracellular oxidants, potentially dangerous for host tissues. Moreover, piceatannol did not affect the phosphorylation of p40^*phox*^—a component of NADPH oxidase, responsible for the assembly of functional oxidase in intracellular (granular) membranes. The stilbenoid tested elevated the percentage of early apoptotic neutrophils, inhibited the activity of protein kinase C (PKC)—the main regulatory enzyme in neutrophils, and reduced phosphorylation of PKC isoforms **α**, **β**II, and **δ** on their catalytic region. The results indicated that piceatannol may be useful as a complementary medicine in states associated with persisting neutrophil activation and with oxidative damage of tissues.

## 1. Introduction

**Figure 1:**
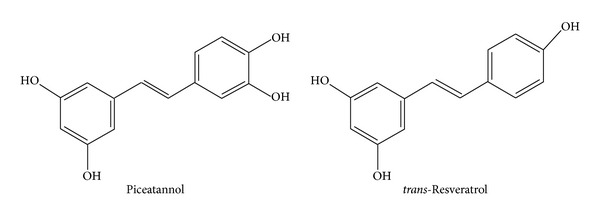
Piceatannol (*trans*-3,4,3′,5′-tetrahydroxystilbene) and its related compound *trans*-resveratrol (*trans*-4,3′,5′-trihydroxystilbene).

Piceatannol (*trans*-3,4,3′,5′-tetrahydroxystilbene, Figure 1) is a naturally occurring hydroxylated analogue of resveratrol, displaying remarkable antioxidative, anticancer, and anti-inflammatory properties. The latter activity has been attributed to the capacity of piceatannol to prevent activation of transcription factors, mRNA expression, and production of inflammatory mediators, for example, nitric oxide, prostaglandin E_2_, interleukins, monocyte chemotactic protein MCP-1, cyclooxygenase-2, and tumour necrosis factor *α* [1–4]. Piceatannol, as a potent spleen tyrosine kinase (Syk) inhibitor, has a great potential to suppress allergic and autoimmune disorders by blocking immune receptor signalling in a variety of inflammatory cells, including neutrophils [5–9].

Neutrophils (neutrophilic polymorphonuclear leukocytes) represent the body's primary line of defense against invading pathogens. Nevertheless, these cells are able to release cytotoxic substances and inflammatory mediators, which, along with their delayed apoptosis, have a potential to maintain permanent inflammation [10, 11]. Therefore, treatment of diseases associated with chronic inflammation should be focused on neutrophil functions; formation of reactive oxygen species (ROS) and apoptosis of these cells represent two promising targets for pharmacological intervention.

Formation of ROS is initiated by the activation of phagocyte NADPH oxidase (NOX2/gp91^*phox*^), the first identified and the best studied member of the NOX family. During activation, the cytosolic oxidase subunits p47^*phox*^, p67^*phox*^, p40^*phox*^, and Rac2 translocate to the plasma membrane and associate with the membrane-bound cytochrome b_558_ complex. Cytochrome b_558_ is formed by two subunits—gp91^*phox*^ (also known as NOX2) and by p22^*phox*^, and this heterodimer transfers electrons from NADPH to molecular oxygen. Flavin adenine dinucleotide (FAD) and two heme groups serve as the redox pathway which enables the transfer of electrons across the membrane [12–14]. If the assembly of catalytically active oxidase occurs on the plasma membrane, the generated oxidants are liberated extracellularly or into phagosomes. These radicals are involved in the elimination of pathogens; however, their overproduction may result in tissue damage. ROS produced intracellularly—on membranes of specific granules, participate in the initiation of neutrophil apoptosis [15, 16] and they are considered key suppressors of inflammation [17, 18]. Since the optimum therapy is expected to minimise tissue damage without reduction of the physiological function of neutrophils, separate analysis of extra- and intracellular effects of antioxidants is of particular importance.

 The production of ROS is limited by the programmed death of neutrophils. Apoptosis represents a precisely regulated process, which includes release of proapoptotic proteins into the cytosol, gradual activation of caspases, DNA fragmentation, chromatin condensation, loss of membrane asymmetry, and formation of apoptotic bodies. Alterations in plasma membrane (e.g., externalisation of phosphatidylserine) facilitate the recognition and clearance of apoptotic neutrophils by macrophages, resulting in safe removal of these cells from the site of inflammation. Since the delayed apoptosis and impaired clearance of neutrophils aggravate and prolong tissue injury, pharmacological intervention focused on neutrophil apoptosis is studied as an original approach for the design of new anti-inflammatory strategies [16, 19, 20]. 

Modulation of protein kinase C activity represents a prospective method to regulate neutrophil functions. Immunochemical studies have shown that human neutrophils express five PKC isoforms, *α*, *β*I, *β*II, *δ*, and *ζ*, which participate in NADPH oxidase activation as well as in proapoptotic and antiapoptotic signalling [21–25].

The present paper investigated the impact of piceatannol on the viability and oxidative burst of human neutrophils. We analysed separately the effects of this stilbenoid on the concentration of ROS produced by neutrophils extra- and intracellularly. Protein kinase C activity was examined as an assumed target of piceatannol action and the phosphorylation of PKC*α*, PKC*β*II, and PKC*δ* (the most abundant PKC isoforms in neutrophils) was assessed.

## 2. Material and Methods

### 2.1. Chemicals

Piceatannol was purchased from Acros Organics (Geel, Belgium). Luminol, isoluminol, PMA (4*β*-phorbol-12*β*-myristate-13*α*-acetate), Ca^2+^-ionophore A23187, superoxide dismutase, dextran (average MW 464 000 kDa), zymosan (zymosan A from *Saccharomyces cerevisiae*), luciferase from *Photinus pyralis*, and D-luciferin sodium salt were from Sigma-Aldrich Chemie (Deisenhofen, Germany); HRP (horseradish peroxidase) and catalase were obtained from Merck (Darmstadt, Germany) and lymphoprep (density 1.077 g/mL) was purchased from Nycomed Pharma AS (Oslo, Norway). Propidium iodide and rh Annexin V-FITC (produced in *E. coli* and conjugated with fluorescein isothiocyanate—FITC) was received from eBioscience (Vienna, Austria) and PKC kinase activity kit was from Enzo Life Sciences AG (Lausen, Switzerland). Phosphospecific antibodies *versus* PKC isoforms and *versus *p40^*phox*^ were obtained from Cell Signaling Technology (Danvers, MA, USA). Secondary anti-rabbit antibody and Lumigen Detection Reagent were supplied by GE Healthcare Life Sciences (Little Chalfont, UK). 

This work was approved by the Local Ethic Committee, Institute of Experimental Pharmacology and Toxicology, Slovak Academy of Sciences, Bratislava. 

### 2.2. Blood Collection and Isolation of Human Neutrophils

Fresh blood was obtained at the blood bank by venipuncture from healthy male donors (20–50 years) who had not received any medication for at least 7 days. The samples were mixed with 3.8% trisodium citrate, in the ratio of 9 mL of blood to 1 mL citrate. Erythrocytes were allowed to sediment in 1% dextran solution (1 ×g, 25 min, 22°C) and the suspension of leukocytes and platelets in plasma (buffy coat) was used for flow cytometric analyses or for neutrophil isolation. For neutrophil isolation, the buffy coat was centrifuged, cells were resuspeded in phosphate-buffered saline, and neutrophils were separated on Lymphoprep (500 ×g, 30 min, 22°C). The contaminating erythrocytes were removed with hypotonic cold haemolysis and neutrophils were washed with phosphate-buffered saline. Neutrophil count was assessed by Coulter Counter (Coulter Electronics, High Wycombe, England) and adjusted to a final concentration of 10^4^ cells/1 *μ*L. The final suspension of neutrophils contained more than 96% of viable cells, as evaluated by trypan blue exclusion, and was used maximally for 2 h—as long as control chemiluminescence kept constant. 

### 2.3. Formation of ROS in Neutrophils

Oxidative burst of neutrophils was recorded on the basis of enhanced chemiluminescence [26, 27], in a microtitre plate computer-driven luminometer LM-01T (Beckman Coulter, Prague, Czech Republic). Chemiluminescence of human whole blood (250x diluted) enhanced with luminol (250 *μ*mol/L) was stimulated with phorbol myristate acetate (PMA, 0.05 *μ*mol/L), opsonized zymosan (0.5 mg/mL), or Ca^2+^-ionophore A23187 (1 *μ*mol/L). Chemiluminescence of isolated human neutrophils (5 × 10^5^/sample) was initiated by PMA (0.05 *μ*mol/L). Oxidants released extracellularly were determined in the system containing isoluminol (5 *μ*mol/L) and HRP (8 U/mL); HRP was added to the system, ensuring sufficient extracellular peroxidase concentration. Intracellular chemiluminescence was enhanced with luminol (5 *μ*mol/L) in the presence of the extracellular scavengers superoxide dismutase (100 U/mL) and catalase (2 000 U/mL). Concentration of oxidants was evaluated on the basis of integral values of chemiluminescence over 1 800 s (isolated neutrophils and A23187 stimulated whole blood) and over 3 600 s (whole blood chemiluminescence initiated with PMA or zymosan).

### 2.4. Chemiluminescence of Cell-Free System

The antioxidative activity of piceatannol was measured in cell-free system containing piceatannol (0.01–100 *μ*mol/L), HRP (2 U/mL), luminol (10 *μ*mol/L), and hydrogen peroxide (100 *μ*mol/L) in 50 *μ*L aliquots. Chemiluminescence was determined for 10 min at 37°C [28]. 

### 2.5. Activity of Protein Kinase C

Isolated neutrophils (5 × 10^5^ cells) were incubated with piceatannol (1–100 *μ*mol/L) for 30 min at 37°C and stimulated with PMA (0.15 *μ*mol/L) for 3 min. Stimulation was stopped by the addition of a tenfold volume of ice-cold phosphate buffer. After centrifugation, neutrophils were broken by lysing solution and by sonication, centrifuged, and the supernatant was used for the determination of protein kinase C activity using an enzyme-linked immunosorbent assay kit.

### 2.6. Phosphorylation of p40^*phox*^ and PKC*α*, *β*II, *δ*

Western blot analysis was performed as previously described [29, 30]. Isolated human neutrophils (5 × 10^6^ cells) were incubated at 37°C with piceatannol (10 or 100 *μ*mol/L, 1 min), stimulated with PMA (0.15 *μ*mol/L, 1 min) and lysed by sonication in a lysing solution containing protease, and phosphatase inhibitors. Debris from the lysed cells was pelleted by centrifugation and the supernatant was taken for blotting assay. Protein concentration was measured using Bradford Dye Reagent detection kit (Bio-Rad, Hercules, CA, USA). Proteins (20 *μ*g per lane) were separated by SDS polyacrylamide gel electrophoresis and transferred to Immobilon-P Transfer Membrane. From each membrane, the area between 60 and 100 kDa was detected with primary anti-phospho-PKC*α*/*β*II (Thr638/641) antibody (1 : 5 000) and with the secondary antibody conjugated to HRP (1 : 5 000). Phosphorylated PKC isoforms *α* and *β*II were visualised with Lumigen Detection Reagent kit, scanned, and quantified densitometrically using ImageJ programme. The membrane was then stripped, using stripping buffer (Re-blot Plus Mild Solution, Millipore, Temecula, CA, USA), and reprobed with anti-phospho-PKC*δ* (Thr505) antibody (1 : 1 000). The membrane area between 30–60 kDa was detected with regard to the presence of the internal standard *β*-actin (*β*-actin antibody, 1 : 4 000) and the phosphorylated subunit of NADPH oxidase p40^*phox*^ (anti-phospho- p40^*phox*^ (Thr154) antibody, 1 : 5 000).

### 2.7. Neutrophil Integrity

Damaging effect of piceatannol on the integrity of plasma membranes was evaluated on the basis of ATP liberation, measured by the luciferin-luciferase chemiluminescence method [31]. Suspension of isolated neutrophils (3 × 10^4^ cells) was incubated with piceatannol (1–100 *μ*mol/L) for 15 min at 37°C. Then, the mixture of luciferin (1.6 *μ*g) and luciferase (45 000 U) was added and chemiluminescence was recorded for 60 seconds. Chemiluminescence of ATP standards (1–500 nmol/L) was measured in each experiment and concentrations of ATP in samples were calculated from the calibration curve. Total ATP content was assessed immediately after sonication of neutrophils for 10 seconds.

### 2.8. Analysis of Apoptosis

Human plasma buffy coat (see Section 2.2) was incubated with piceatannol (1–100 *μ*mol/L) for 10 min at 37°C. The cells were double-stained with annexin-V conjugated with FITC (in dark at 4°C for 10 min) and with propidium iodide and analysed by cytometer Cytomics FC 500 (Beckman Coulter, Inc., Brea, CA, USA). From the granulocyte area, 5 000 cells were gated and the percentage of apoptotic (annexin positive and propidium iodide negative), dead (double positive), and viable cells (double negative) was determined as described previously [32, 33]. 

### 2.9. Data Analysis

All values were given as the mean ± SEM and the statistical significance of differences between means was established by Student's *t*-test. *P* values below 0.05 were considered to be statistically significant and were indicated in the figures by *P ≤ 0.05 and **P ≤ 0.01.

## 3. Results

Piceatannol reduced the oxidative burst of human neutrophils measured in whole blood (Table 1). It inhibited chemiluminescence initiated by the stimulation of protein kinase C, increased calcium concentration, and the activation of membrane receptors at the respective mean effective concentrations of 0.65 ± 0.07 *μ*mol/L (PMA), 2.71 ± 0.41 *μ*mol/L (A23187) and 9.43 ± 0.53 *μ*mol/L (zymosan).

**Figure 2:**
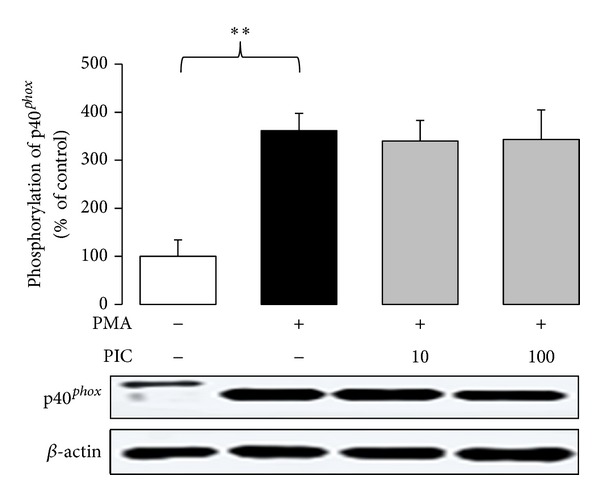
Phosphorylation of p40^*phox*^ in PMA-stimulated neutrophils treated with 10 and 100 *μ*mol/L piceatannol. Phosphorylated NADPH oxidase subunit was isolated by Western blotting and detected with anti-phospho- p40^*phox*^ (Thr154) antibody. The values are presented as percentage of resting control. Control value, given as optical density of p40^*phox*^ band corrected to *β*-actin content, was 38.13 ± 10.67. Mean ± SEM, *n* = 6, **P ≤ 0.01.

**Figure 3:**
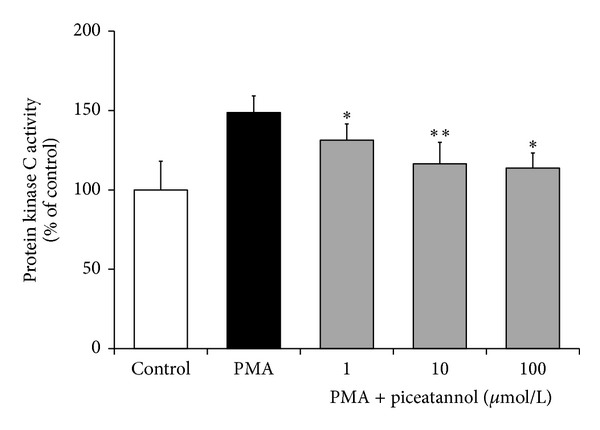
. Effect of piceatannol on PKC activity. Neutrophils were incubated with piceatannol (30 min) and stimulated with PMA (3 min). PKC activity was assessed by ELISA kit in the supernatant of cell lysate. The values are presented as percentage of resting control (PKC activity in absence of PMA). Control value given as kinase activity per 1 mg of protein was 13 376 ± 2 417. Mean ± SEM, *n* = 5, *P ≤ 0.05, **P ≤ 0.01  *versus* PMA.

**Figure 4:**
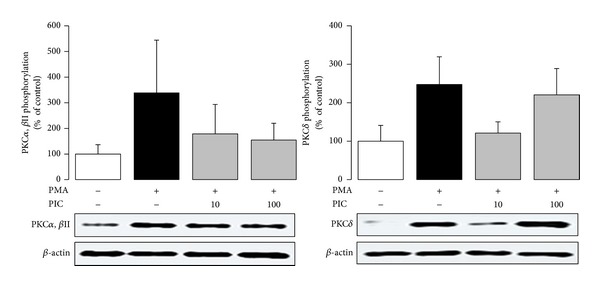
Phosphorylation of PKC*α*, PKC*β*II, and PKC*δ* in PMA-stimulated neutrophils treated with 10 and 100 *μ*mol/L piceatannol (PIC). Phosphorylated PKC isoforms were isolated by Western blotting and detected with anti-phospho-PKC*α*/*β*II (Thr638/641) and anti-phospho-PKC*δ* (Thr505) antibodies. The values are presented as percentage of resting control. Control values, given as optical density of PKC bands corrected to *β*-actin content, were 78.07 ± 17.86 (PKC*α*, *β*II) and 84.84 ± 18.80 (PKC*δ*). The representative blot manifests elevated phosphorylation of PKC isoforms in neutrophils stimulated with PMA as well as the effect of piceatannol on this increase. Mean ± SEM, *n* = 4.

**Figure 5:**
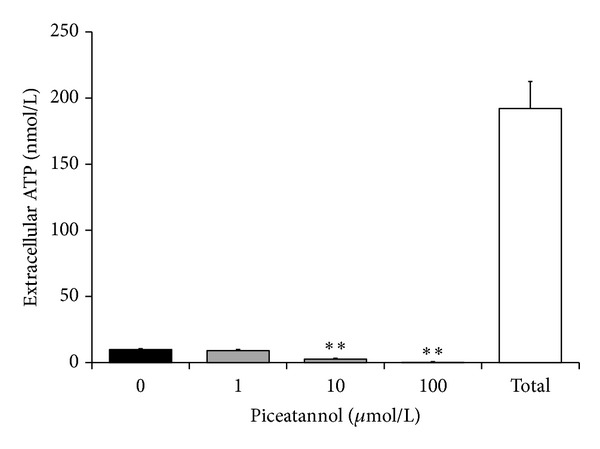
Integrity of neutrophils assessed on the basis of ATP liberation in the absence (0) and in the presence of piceatannol (1–100 *μ*mol/L). Total—amount of ATP determined immediately after complete neutrophil destruction. The given values represent the extracellular ATP concentration in samples containing 30 000 neutrophils. Mean ± SEM, *n* = 6, **P ≤ 0.01  *versus* Control (0).

**Figure 6:**
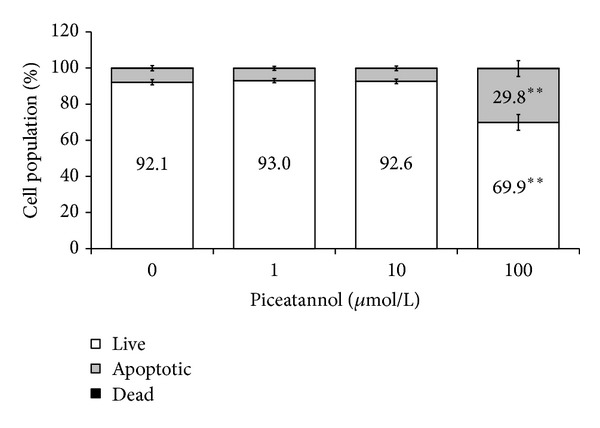
Effect of piceatannol on neutrophil apoptosis. The lifespan of human neutrophils was recorded by flow cytometry, using double-staining with annexin-V (AN) and propidium iodide (PI). Of the population of 5 000 granulocytes, the percentage of live (AN^−^/PI^−^), apoptotic (AN^+^/PI^−^), and dead cells (AN^+^/PI^+^) was calculated. The percentage of dead neutrophils (0.1-0.2%) was not significantly increased in the presence of piceatannol. Mean ± SEM, *n* = 7, **P ≤ 0.01  *versus* Control (0).

In isolated neutrophils stimulated with PMA, extra- and intracellular chemiluminescence was recorded separately (Table 2). Piceatannol decreased both the extracellular and intracellular chemiluminescence of neutrophils at the respective mean effective concentrations 1.87 ± 0.35 *μ*mol/L and 12.59 ± 0.96 *μ*mol/L; in cell-free system, the EC_50_ of piceatannol was 0.63 ± 0.01 *μ*mol/L. The phosphorylation of p40^*phox*^ (a component of NADPH oxidase essential for intracellular oxidant formation) was increased more than three times after PMA stimulation. This increase was not modified by the treatment of neutrophils with piceatannol (Figure 2). Considering the high efficiency of piceatannol in neutrophils stimulated with PMA and its recorded intracellular activity, in further experiments the effect of this phytochemical was evaluated on PKC activity (Figure 3). The stimulation of neutrophils with PMA increased protein kinase C activity by 50%; piceatannol dose-dependently reduced this rise until the values of activity were comparable with those produced by resting cells. The phosphorylation of protein kinases C *α*, *β*II, and *δ* (the most abundant PKC isoforms in neutrophils) was also decreased after piceatannol treatment (Figure 4). Phosphorylation of PKC*α* and *β*II was reduced in the presence of both concentrations used, whereas in the case of PKC*δ* phosphorylation, only 10 *μ*mol/L piceatannol was effective. The observed inhibitory effects were not associated with neutrophil damage as in the presence of piceatannol no increase in extracellular ATP concentration was recorded (Figure 5). Spontaneous ATP liberation from isolated neutrophils was minimal, approximately 5% of the total ATP content. This amount remained unchanged or was slightly decreased after treatment of neutrophils with piceatannol. Spontaneous neutrophil apoptosis was accelerated by piceatannol as indicated by the significantly elevated number of apoptotic neutrophils (Figure 6). The percentage of dead (propidium iodide positive) neutrophils ranged from 0.1 to 0.2% and was not significantly increased in the presence of piceatannol.

## 4. Discussion

Novel strategies of anti-inflammatory therapy are based upon pharmacological agents capable of enhancing the resolution—that is, the termination of the beneficial inflammation before it may turn into an adverse chronic stage [34, 35]. It is likely that several phytochemicals would act in this way, but this point has not been investigated. In this paper, the natural stilbenoid piceatannol was analysed, considering its ability to affect activity and apoptosis of neutrophils—two important inputs of resolution [19, 20, 36]. 

The incubation of human neutrophils with piceatannol resulted in decreased production of ROS. Several mechanisms—inhibition of Syk kinase, ROS scavenging, and the interference with neutrophil activation through suppressed PKC activation—could be involved in the reduction of the chemiluminescence signal. Piceatannol, widely used as an inhibitor of Syk kinase [6, 8, 9], has the potential to block oxidative burst at the receptor signaling level. However, this mechanism might not be essential, with regard to the pronounced inhibition of chemiluminescence initiated with PMA or A23187, that is, by receptor bypassing stimuli. The marked inhibition of the chemiluminescence produced by cell-free system suggested participation of antioxidant activity, which is closely involved in various effects of other natural polyphenols [37–41]. Piceatannol was found to be a potent scavenger of hydroxyl radicals and superoxide anion [42], much more effective than resveratrol. This high effectiveness results from the presence of an additional hydroxyl group in stilbene rings, which makes the abstraction and transfer of electrons easier and increases the stability of the resulting piceatannol semiquinone radical [4]. Due to the fact that the inhibition of chemiluminescence occurred in the presence of each stimulus used, the interference of piceatannol with a process involved in all mechanisms of initiation has been suggested. One of the potential candidates could be the signalling enzyme protein kinase C. Piceatannol inhibited PKC activation initiated by PMA, which was reflected in the decreased activity of the enzyme as well as in the reduced phosphorylation of protein kinase isoenzymes *α*, *β*II, and *δ* on their catalytic region. Since PKC participates in the activation of neutrophil NADPH oxidase [13, 14, 22, 25], its inhibition may result in reduced oxidant formation and thus explain the decreased chemiluminescence of neutrophils treated with piceatannol. The precise mechanism of piceatannol-mediated PKC inhibition is still not completely clear. Similar to other natural polyphenols, it may involve the competition for phorbol ester or calcium binding to the regulatory domains of PKC [43, 44], inhibition of PKC translocation from cytosol to membrane fraction [45], oxidation of thiol groups present within the catalytic domain of PKC [46], or piceatannol-induced alterations in membrane ordering [47].

Since activated neutrophils form and liberate ROS both extra- and intracellularly [13, 17, 18], it was important to identify which part of the chemiluminescence signal was reduced in the presence of piceatannol. This stilbenoid was active in both compartments, however, at different mean effective concentrations −12.59 *μ*mol/L (intracellular) and 1.87 *μ*mol/L (extracellular). It means that the radicals formed within neutrophils (fulfilling a regulatory role) were reduced to a lesser extent than extracellular oxidants, potentially dangerous for host tissues. Moreover, piceatannol did not affect the phosphorylation of p40^*phox*^—a component of NADPH oxidase, responsible for the assembly of functional oxidase in intracellular (granular) membranes [17, 48, 49]. Finally, the phosphorylation of PKC*δ* (responsible for intracellular oxidant production [50]) was affected by piceatannol to a lesser extent than the phosphorylation of PKC*β*, which is involved in the extracellular formation of oxidants [16]. Yet the interference of piceatannol with PKC*δ* may not be a decisive mechanism involved in the inhibition of intracellular chemiluminescence, as in the presence of 100 *μ*mol/L piceatannol, the chemiluminescence was strongly reduced, despite the fact that the inhibition of PKC*δ* phosphorylation was minimal at this concentration.

The more pronounced extracellular activity results from the structure of piceatannol. Compared to resveratrol, the additional hydroxyl group makes the molecule of piceatannol more hydrophilic and less able to pervade biological membranes [47]. Thus, piceatannol could minimise tissue damage with the minimal reduction of beneficial intracellular oxidants, involved in the suppression of neutrophil proinflammatory activity [17, 18] and in the initiation of neutrophil apoptosis [15, 16]. 

The observed reductions in chemiluminescence and in the activity of protein kinase C were not associated with neutrophil damage because in the presence of piceatannol no increase in extracellular ATP concentration was recorded. As confirmed by flow cytometry, this stilbene enhanced spontaneous apoptosis of neutrophils. This was indicated by an increased number of annexin-positive cells, that is, cells displaying a more pronounced externalisation of phosphatidylserine. The expression of phosphatidylserine on the external side of plasma membrane facilitates the recognition of apoptotic neutrophils by macrophages and their safe removal from the site of inflammation [15]. The ability of piceatannol to increase apoptosis has been extensively studied in cancer cells, where it involves the increased activities of caspases, activation of proapoptotic factors Bid, Bax, Bak, or the inhibition of the antiapoptotic factor Bcl-xL [4]. In neutrophils, all these mechanisms may be operative, along with the repressed activation of the antiapoptotic enzyme phosphoinositide-3-kinase [51, 52]. Moreover, the accelerated apoptosis, observed in the presence of piceatannol, may result from the inhibition of PKC*α* and PKC*δ*, as these PKC isoforms participate in antiapoptotic signalling in neutrophils [23, 53]. Increased neutrophil apoptosis was observed in the presence of 100 *μ*mol/L piceatannol, that is, in a concentration several times higher than assumed piceatannol plasma levels obtained by dietary intake. Yet it should be taken into account that, in comparison to *in vivo* conditions, in these samples neutrophil count was substantially higher and the exposure of the cells to piceatannol lasted only 30 minutes. Similarly, the accelerated apoptosis of leukemic cells was detected after 24–48 h incubation with 10–60 *μ*mol/L piceatannol [54, 55].

## 5. Conclusion

Piceatannol decreased the concentration of ROS produced by neutrophils and accelerated spontaneous apoptosis of these cells. The observed effects classified piceatannol as a potentially useful complementary medicine in states associated with persistent activation of neutrophils, oxidative damage of tissues, and persistent inflammation. However, the bioavailability and toxicity of this promising natural substance is a decisive question requiring further studies.

## Figures and Tables

**Table 1:** Dose-dependent inhibition of neutrophil oxidative burst in the presence of piceatannol. Chemiluminescence, measured in whole blood, was initiated with PMA (0.05 *μ*mol/L), Ca^2+^-ionophore A23187 (1 *μ*mol/L), or opsonized zymosan (0.5 g/L). The incubation of neutrophils with piceatannol was 60 min (PMA, zymosan) or 30 min (A23187), depending on the kinetics of oxidative burst. Mean ± SEM, *n* = 8, *P ≤ 0.05, **P ≤ 0.01  *versus* Control.

Piceatannol (*µ*mol/L)	Inhibition of chemiluminescence (% of control)		
	PMA	A23187	Zymosan
0.01	93.21 ± 2.64*	96.74 ± 1.41	98.10 ± 1.10
0.1	84.45 ± 1.99**	91.64 ± 1.84**	98.63 ± 1.42
1	41.19 ± 2.19**	73.89 ± 4.39**	93.22 ± 1.98*
10	4.12 ± 0.21**	19.40 ± 3.30**	50.17 ± 1.37**
100	0.25 ± 0.04**	0.07 ± 0.07**	0.26 ± 0.07**

**Table 2:** Effect of piceatannol on extra- and intracellular chemiluminescence of isolated human neutrophils stimulated with PMA (0.05 *μ*mol/L) and on the chemiluminescence produced by cell-free system. The exposure to piceatannol was 30 min (extra-, intracellular) or 10 min (cell-free system). Mean ± SEM, *n* = 3–8, *P ≤ 0.05, **P ≤ 0.01  *versus* Control.

Piceatannol (*µ*mol/L)	Inhibition of chemiluminescence (% of control)		
	Extracellular	Intracellular	Cell-free system
0.01	96.89 ± 4.05	101.72 ± 4.34	99.43 ± 1.19
0.1	90.73 ± 5.80	102.40 ± 2.16	93.77 ± 0.55*
1	75.74 ± 5.55*	101.29 ± 1.96	33.60 ± 0.13**
10	5.31 ± 0.82**	59.95 ± 2.51**	0.72 ± 0.04**
100	0.01 ± 0.01**	0.65 ± 0.15**	0.71 ± 0.02**
